# The discrepancy among single nucleotide variants detected by DNA and RNA high throughput sequencing data

**DOI:** 10.1186/s12864-017-4022-x

**Published:** 2017-10-03

**Authors:** Yan Guo, Shilin Zhao, Quanhu Sheng, David C Samuels, Yu Shyr

**Affiliations:** 1 Department of Biomedical Informatics, Vanderbilt University, 2220 Pierce Ave, 571 PRB, Nashville, TN 37027 USA; 2 Vanderbilt Genetics Institute, Department of Molecular Physiology and Biophysics, Vanderbilt University Medical School, Nashville, TN USA; 3 Department of Biostatistics, Vanderbilt University, 2220 Pierce Ave, 571 PRB, Nashville, TN 37027 USA

**Keywords:** DNA-RNA difference, RNA editing, Single nucleotide variant

## Abstract

**Background:**

High throughput sequencing technology enables the both the human genome and transcriptome to be screened at the single nucleotide resolution. Tools have been developed to infer single nucleotide variants (SNVs) from both DNA and RNA sequencing data. To evaluate how much difference can be expected between DNA and RNA sequencing data, and among tissue sources, we designed a study to examine the single nucleotide difference among five sources of high throughput sequencing data generated from the same individual, including exome sequencing from blood, tumor and adjacent normal tissue, and RNAseq from tumor and adjacent normal tissue.

**Results:**

Through careful quality control and analysis of the SNVs, we found little difference between DNA-DNA pairs (1%–2%). However, between DNA-RNA pairs, SNV differences ranged anywhere from 10% to 20%.

**Conclusions:**

Only a small portion of these differences can be explained by RNA editing. Instead, the majority of the DNA-RNA differences should be attributed to technical errors from sequencing and post-processing of RNAseq data. Our analysis results suggest that SNV detection using RNAseq is subject to high false positive rates.

**Electronic supplementary material:**

The online version of this article (doi:10.1186/s12864-017-4022-x) contains supplementary material, which is available to authorized users.

## Background

Single nucleotide variants (SNVs) are often measured in human specimens to correlate with other phenotypic variables. In general, there are two major classes of SNVs: germline mutations, which are inherited with one allele from each parent (also known as germline), and somatic mutations which are acquired at late stage of life. Germline mutations are usually used to assess the risk of developing certain diseases. Somatic mutations are often associated with tumorigenesis. Both germline and somatic mutations have been studied extensively in biomedical research. Single nucleotide polymorphisms (SNPs) describe germline mutations at population level.

The detection of SNVs can be achieved through a variety of methods, including real time polymerase chain reaction (RT-PCR), genotyping array, Sanger sequencing, and high throughput sequencing. All of these methods use genomic DNA as the input source. For example, genome wide association studies (GWAS) typically use DNA extracted from blood to infer SNPs due to the easy collection and storage of blood. Somatic mutations occur in tumor tissues and are usually identified by comparing the DNA sequences of tumor tissue to blood or adjacent normal tissue. One of the most basic assumptions of human DNA is that in the absence of somatic mutations, every cell in the body is essentially identical. A study in 2009 by Gottlieb et al. challenged this conventional paradigm by identifying three SNPs in tissues that were not present in blood [1]. This finding received great attention, while simultaneously receiving criticism for the inaccuracy of the analyses [2].

High throughput sequencing technology enables investigators to screen for SNVs in the entire genome or exome at a reasonable cost. Over the same time period, the development of RNAseq technology has replaced microarrays as the primary tool for gene expression profiling [3–6]. Unlike microarrays, RNAseq is based on high throughput sequencing technology, and thus investigators can now also examine RNA genomic sequences at a single nucleotide resolution. RNAseq technology introduces an opportunity to compare the genomic sequences of DNA and RNA at an unprecedented large scale.

RNAseq data is often thought to be a less-than-ideal source for SNV detection due to higher false positive rates [7]. The higher false positive rates can be attributed to several reasons, including higher complexity in alignment due to the RNA splicing [8], random errors introduced during reverse transcription, PCR [9] and RNA editing [9]. Numerous attempts have been made to overcome these difficulties [8, 10, 11] with only moderate success.

The differences between DNA and RNA sequences have been previously documented. For example, Li et al. [12] reported that they observed widespread differences between the RNA and DNA sequences of the same human cells. Since its publication, three other independent follow-up studies [13–15] challenged the conclusion by Li et al., arguing that the differences found by Li et al. are attributed to alignment artifacts, RNA editing, etc. To address this controversy, a more in-depth analysis of sequence data is required to discern the true differences between RNA and DNA sequences.

To date, there is no clear consensus on how much genomic difference we should expect to see between blood and tissue, and between DNA and RNA of the same subject. Answering these questions can greatly contribute to the accuracy of SNV and somatic mutation identification from multiple sources.

## Methods

To fully understand the single nucleotide differences between blood-tissue and DNA-RNA pairs, we conducted a thorough study that compared the nucleotide sequences between each sample-sequencing pair type, using a unique set of sequencing data from TCGA. From TCGA, we obtained sequencing data of 50 samples from 10 breast cancer patients. Each patient had five samples collected and sequenced: 1) DNA exome sequencing on blood (DNA-NB), 2) DNA exome sequencing on tumor primary tissue (DNA-TP), 3) DNA exome sequencing on adjacent normal tissue (DNA-NT), 4) RNAseq on tumor primary tissue (RNA-TP), 5) RNAseq on adjacent normal tissue (RNA-NT). Data for all samples were downloaded from the Cancer Genomics Hub in aligned BAM format [16]. Since the DNAseq data and RNAseq data were processed by different facilities, to ensure data integrity, we converted all BAM files to raw FASTQ formats and performed alignment against the human genome reference (HG19) using BWA [17] for DNAseq data and TopHat 2 [18] for RNAseq data. The alignment statistics can be found in Table 1. Next, we marked duplicates using Picard [19], then performed local realignment and local recalibration using the Genome Analysis Toolkit (GATK) [20] developed by the Broad Institute.

**Table 1:** Alignment summary

Sample	Total reads	Mapped reads	Unmapped reads
TCGA-A7-A0D9-DNA_NB	142,860,012	136,174,786	6,685,226
TCGA-A7-A0D9-DNA_NT	158,844,243	155,372,460	3,471,783
TCGA-A7-A0D9-DNA_TP	138,383,452	136,764,896	1,618,556
TCGA-A7-A0D9-RNA_NT	141,376,864	134,152,483	7,224,381
TCGA-A7-A0D9-RNA_TP	149,200,610	141,630,167	7,570,443
TCGA-BH-A0B3-DNA_NB	211,311,809	209,315,407	1,996,402
TCGA-BH-A0B3-DNA_NT	170,360,878	165,875,755	4,485,123
TCGA-BH-A0B3-DNA_TP	159,731,541	158,253,223	1,478,318
TCGA-BH-A0B3-RNA_NT	164,452,329	156,498,369	7,953,960
TCGA-BH-A0B3-RNA_TP	164,079,925	155,833,920	8,246,005
TCGA-BH-A0B8-DNA_NB	171,951,966	170,021,858	1,930,108
TCGA-BH-A0B8-DNA_NT	143,464,049	140,389,068	3,074,981
TCGA-BH-A0B8-DNA_TP	216,218,230	213,713,105	2,505,125
TCGA-BH-A0B8-RNA_NT	152,562,120	143,571,886	8,990,234
TCGA-BH-A0B8-RNA_TP	128,002,634	122,243,065	5,759,569
TCGA-BH-A0BJ-DNA_NB	147,410,868	145,768,369	1,642,499
TCGA-BH-A0BJ-DNA_NT	162,172,150	158,678,527	3,493,623
TCGA-BH-A0BJ-DNA_TP	143,442,013	141,770,778	1,671,235
TCGA-BH-A0BJ-RNA_NT	138,807,984	131,847,427	6,960,557
TCGA-BH-A0BJ-RNA_TP	149,966,756	144,440,232	5,526,524
TCGA-BH-A0BM-DNA_NB	159,310,853	156,835,192	2,475,661
TCGA-BH-A0BM-DNA_NT	165,501,253	162,838,285	2,662,968
TCGA-BH-A0BM-DNA_TP	119,192,355	117,149,967	2,042,388
TCGA-BH-A0BM-RNA_NT	149,007,565	138,576,725	10,430,840
TCGA-BH-A0BM-RNA_TP	117,977,848	100,498,089	17,479,759
TCGA-BH-A0C0-DNA_NB	176,208,298	173,440,163	2,768,135
TCGA-BH-A0C0-DNA_NT	177,261,968	172,796,230	4,465,738
TCGA-BH-A0C0-DNA_TP	143,339,652	141,217,919	2,121,733
TCGA-BH-A0C0-RNA_NT	189,543,380	180,211,183	9,332,197
TCGA-BH-A0C0-RNA_TP	125,992,620	118,740,948	7,251,672
TCGA-BH-A0DK-DNA_NB	160,749,783	158,782,935	1,966,848
TCGA-BH-A0DK-DNA_NT	158,654,513	155,523,188	3,131,325
TCGA-BH-A0DK-DNA_TP	178,103,631	175,051,156	3,052,475
TCGA-BH-A0DK-RNA_NT	191,328,391	184,115,083	7,213,308
TCGA-BH-A0DK-RNA_TP	143,488,953	136,143,128	7,345,825
TCGA-BH-A0DP-DNA_NB	157,712,348	155,347,716	2,364,632
TCGA-BH-A0DP-DNA_NT	167,557,587	163,348,435	4,209,152
TCGA-BH-A0DP-DNA_TP	168,097,321	165,554,381	2,542,940
TCGA-BH-A0DP-RNA_NT	169,655,182	159,641,615	10,013,567
TCGA-BH-A0DP-RNA_TP	136,210,380	129,171,483	7,038,897
TCGA-BH-A0E0-DNA_NB	151,357,163	141,201,519	10,155,644
TCGA-BH-A0E0-DNA_NT	159,040,104	156,614,176	2,425,928
TCGA-BH-A0E0-DNA_TP	130,825,456	129,444,757	1,380,699
TCGA-BH-A0E0-RNA_NT	146,561,149	136,899,519	9,661,630
TCGA-BH-A0E0-RNA_TP	111,749,610	105,126,617	6,622,993
TCGA-BH-A0H7-DNA_NB	170,784,467	168,285,144	2,499,323
TCGA-BH-A0H7-DNA_NT	173,665,210	169,363,318	4,301,892
TCGA-BH-A0H7-DNA_TP	156,659,296	154,959,185	1,700,111
TCGA-BH-A0H7-RNA_NT	154,651,936	146,599,114	8,052,822
TCGA-BH-A0H7-RNA_TP	186,962,558	179,990,244	6,972,314

Genotypes were inferred by HaplotypeCaller from GATK. GATK best practice filters were used to filter out potential false positive SNPs. For the five sample-sequencing types—DNA-NB, DNA-NT, DNA-TP, RNA-NT, and RNA-TP—there are 10 possible pairs. For each pair of samples, we computed the heterozygous genotype consistency as follows: the number of consistent heterozygous genotypes between sample A and sample B divided by the total number of heterozygous genotypes in sample A or in sample B. We only considered genomic positions covered with at least 10 reads in both samples of the pair (denoted as “callable” sites). Also, we focused our study on haploid genomes (chromosome 1–22). Chromosome X, Y and mitochondrial DNA were not considered in this study.

**Fig. 1:**
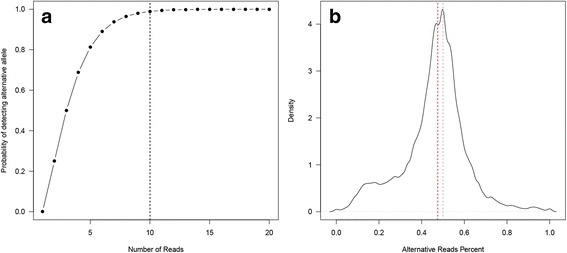
**a** The probability of detecting the alternative allele given depth under the binomial distribution. **b** The distribution of the allele frequency for alternative allele. The expected value is 0.5, the actual median measure is a few percent shifted to the left (*red dotted line*), caused by reference preferential biases

The identification of an alternative allele at a certain genomic location is highly dependent on the depth of coverage. For a heterozygous position, the reads that support the alternative allele should ideally follow a binomial distribution,*Binomial*(*D*, 0.5). Thus, we expect to observe an alternative allele at 50% allele frequency. The probability of observing an alternative allele increases as the depth increases (Fig. 1a). As seen from this figure, setting the depth threshold at 10 allows a higher probability to observe an alternative allele. However, the distribution of the alternative-allele frequency among the reads produced by a sequencing dataset usually follows a normal distribution (Fig. 1b). Thus, there are some genotypes with extremely high or low allele frequencies that deviate from 50%. Also, during sequencing alignment, reference preferential biases can also skew the distribution of allele frequencies by 2 to 5% toward the reference allele [21]. Reference preferential bias is a type of alignment artifact, since most aligners will penalize the alignment of a read based on the number of mismatches within that read. A true SNP is counted as a mismatch during alignment, thus the aligner preferentially prefers reads with no mismatches in the alignment and slightly undercounts the reads containing alternate alleles. Furthermore, four of our five sample types are extracted from tissues in or around the tumor with acquired somatic mutation, which may contain some tumor cells carrying somatic mutaitons. For these reasons, the percentage of mutated alleles at a genomic location does not strictly follow the *Binomial*(*D*, 0.5) distribution. It is possible that only a small percentage of reads support mutated alleles. To take this into consideration, we also computed a loose genotype consistency between a pair of samples. The loose genotype consistency is computed in the same way as described above, with the exception that consistent heterozygous genotypes between samples A and B are defined as the genotypes that are consistent if they have the same alternative allele supported by at least one read that passed the quality filter (base quality >20). Thus, the actual genotype call by the HaplotypeCaller is irrelevant in this calculation.

We studied the pattern of potential RNA editing by examining the flanking sequences of the different RNA-DNA sites and the frequency of the RNA-DNA nucleotide change types. Motif analysis was carried out using HOMER [22]; cluster analysis was performed using Heatmap3 [23]. Additional annotation on the different RNA-DNA sites was done using previously reported editing sites, as described in the databases RADAR [24] and DARNED [25].

## Results

**Fig. 2:**
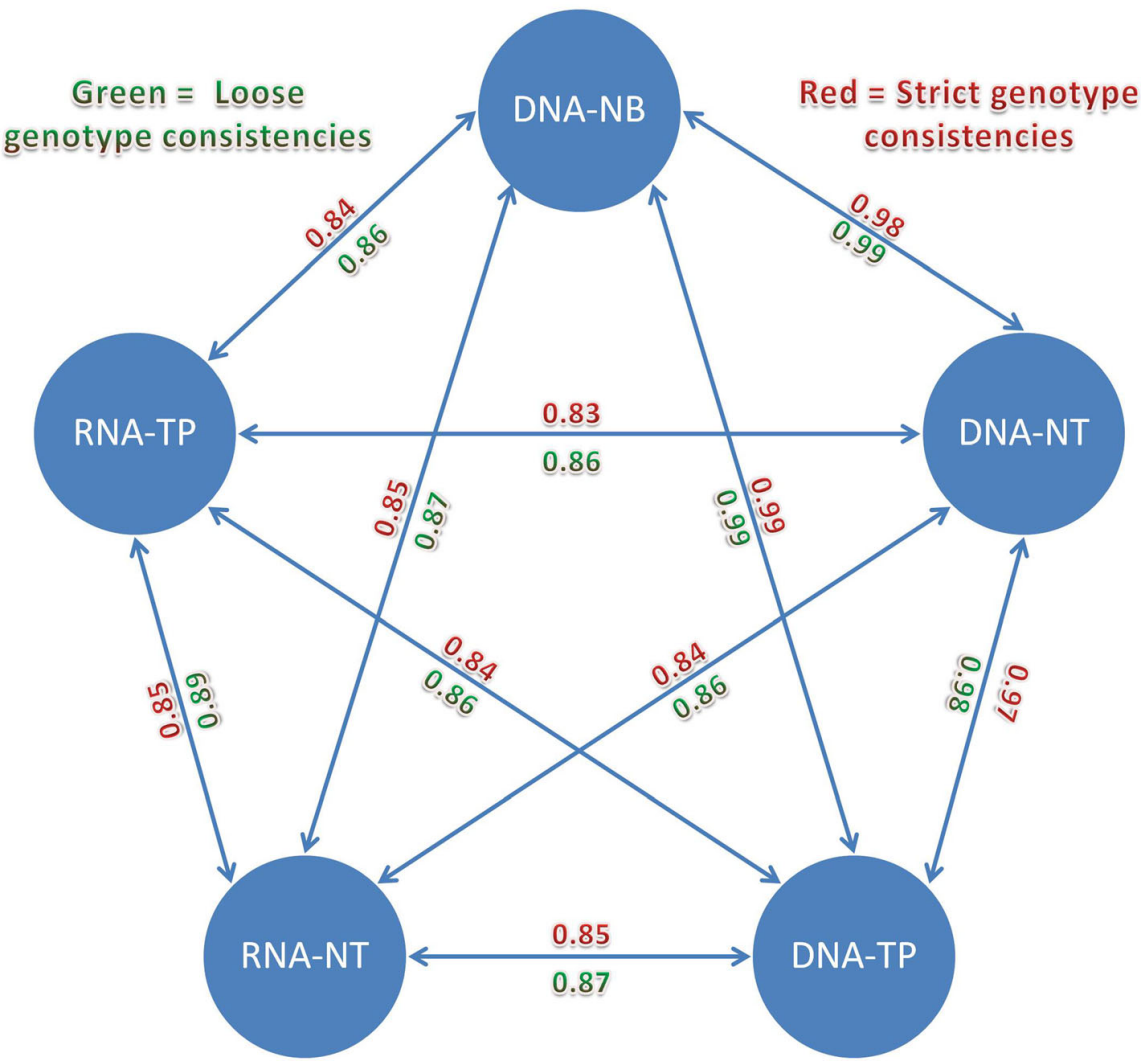
Genotype consistencies between any two pairs of sequencing data

The heterozygous consistency analysis results showed high consistency rates (0.96–0.99) (Fig. 2, Table 2) between sequencing data pairs of DNA samples from all three tissue sources. After RNA data was introduced into the pairings, the heterozygous consistency rate dropped substantially (0.79–0.90). As expected, the loose heterozygous consistency is higher in comparison to the regular heterozygous consistency, achieving a range of 0.97–0.99 for DNA-DNA pairs, and a range of 0.82–0.91 for DNA-RNA pairs. Due to the occurrence of errors and noise during library preparation, sequencing, and/or alignment, there will always be nucleotide differences even between DNA sequencing data of technically-replicated samples. The minor differences observed between DNA-NB and DNA-NT can also be contributed to tumor contamination in the adjacent normal tissue. The large differences observed between the DNA-RNA pairs confirm previous findings that large amounts (10–21%) of callable nucleotides are observed differently in DNA as compared to RNA sequencing data. We demonstrate the DNA-RNA difference using one example by Integrative Genomics Viewer (Additional file 1).

**Table 2:** Heterozygous genotype consistencies

		Strict^a^		Loose^b^	
Sample A	sample B	Consistency A^c^	Consistency B^d^	Consistency A^c^	Consistency B^d^
DNA-NB	DNA-NT	0.99	0.97	0.99	0.98
DNA-NB	DNA-TP	0.98	0.99	0.99	0.99
DNA-NB	RNA-NT	0.90	0.80	0.91	0.83
DNA-NB	RNA-TP	0.84	0.83	0.87	0.85
DNA-NT	DNA-TP	0.96	0.98	0.97	0.99
DNA-NT	RNA-NT	0.89	0.79	0.90	0.82
DNA-NT	RNA-TP	0.82	0.83	0.86	0.85
DNA-TP	RNA-NT	0.90	0.79	0.91	0.82
DNA-TP	RNA-TP	0.84	0.83	0.87	0.85
RNA-NT	RNA-TP	0.82	0.87	0.86	0.91

^a^Strict means if two genotypes are consistent, their genotype call from Unifiedgenotyper has to agree

^b^Loose means if two genotypes are consistent, their alternative alleles has to be supported by at least 1 read with BQ > 20 at that position

^c^The genotype consistency is computed with the number of heterozygous genotypes of sample A as denominator

^d^The genotype consistency is computed with the number of heterozygous genotypes of sample B as denominator

**Fig. 3:**
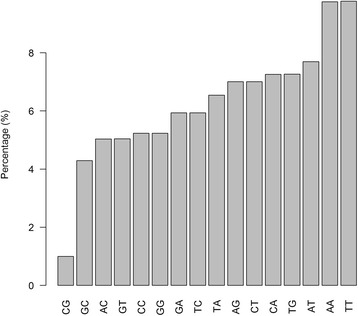
Background dinucleotide distribution computed from GRCh37

**Fig. 4:**
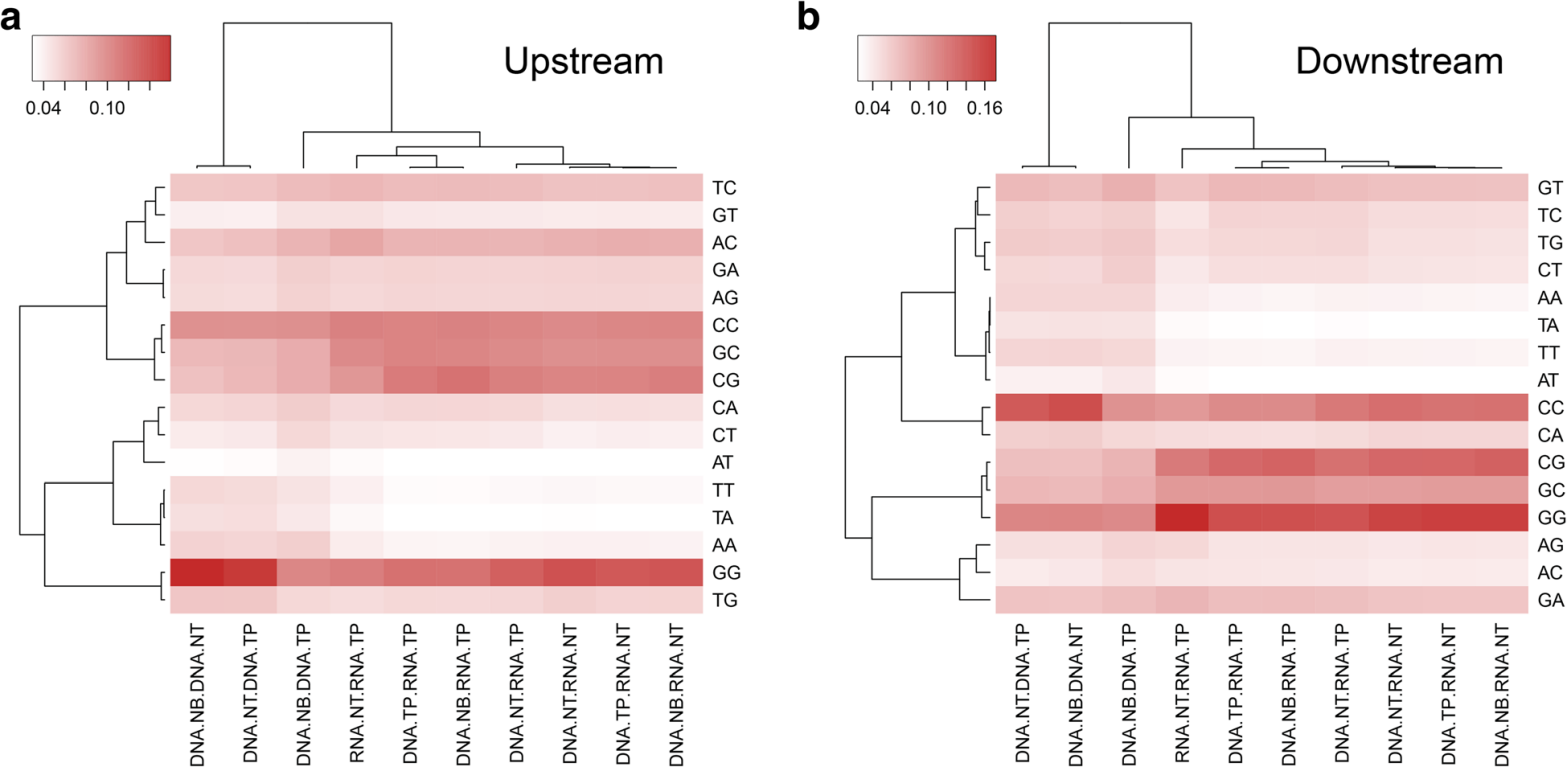
Cluster and heatmaps based on upstream and downstream dinucleotide patterns. Clear differentiation can be observed based on whether RNA is included in the comparisons

We performed dinucleotide distribution analysis. First, we computed the global dinucleotide frequencies for the human genome (Fig. 3). The most preferred dinucleotide is TT (9.78%) and AA (9.75%), and the least preferred dinucleotide is CG (1.00%), followed by GC (4.29%). For all of the SNV differences that we observed between any sample-sequencing type of the same subject, we extracted the up- and down-stream dinucleotides of the site, then we normalized them to the human genome background dinucleotide frequencies. Clean patterns emerged when we used the normalized dinucleotide frequencies in cluster analysis. For the two nucleotides upstream and downstream of the discordant genotype sites, two major clusters were formed by sample-sequence types: a smaller cluster containing two pairs involving only DNA samples, and a larger cluster containing eight pairs, in which seven involve RNA samples (Fig. 4). The most preferred dinucleotides both up- and down-stream were GG (upstream: 12%, downstream: 14%), followed by CC (upstream: 10%, downstream: 11%). These findings suggest GC content plays a role in the mismatched genotypes. The least preferred dinucleotide for both upstream and downstream was AT (upstream: 3.1%, downstream: 2.9%). For the upstream two nucleotides, pairs with RNAseq data had higher frequencies for CC (t test *p* = 1.03E-5) and CG (t test *p* = 0.001), while pairs with only DNA sequencing data had higher frequencies in AA (t test *p* = 0.0008) and TT (t test *p* = 0.002). For the downstream two nucleotides, pairs with RNAseq data had higher frequencies for GG (t test *p* = 7.56 × 10^−6^) and CG (t test *p* = 1.41 × 10^−5^), while pairs with only DNA sequencing data had higher frequencies in AA (t test *p* = 2.25 × 10^−8^) and TA (t test *p* = 2.52 × 10^−8^). The detailed allele frequencies and analysis are presented in Tables 3 and 4. Next, we examined whether or not a pattern could be observed in the DNA-RNA difference. We took 10 nucleotides up and downstream of the DNA-RNA difference sites and identified no significant motif using Homer [22].

**Table 3:** Upstream dinucleotide distribution

Dinucleotide	DNA NB DNA NT	DNA NB DNA TP	DNA NT DNA TP	DNA NB RNA NT	DNA NB RNA TP	DNA NT RNA NT	DNA NT RNA TP	DNA TP RNA NT	DNA TP RNA TP	RNA NT RNA TP
CC	9.37%	9.45%	9.33%	10.05%	10.29%	9.84%	10.07%	10.00%	10.17%	10.33%
AA	5.57%	5.78%	5.37%	3.77%	3.65%	3.89%	3.82%	3.88%	3.73%	4.14%
CG	6.54%	7.71%	7.04%	10.51%	11.27%	10.11%	10.39%	10.16%	10.69%	9.03%
GC	6.97%	7.73%	7.14%	9.53%	10.06%	9.47%	9.78%	9.54%	10.17%	9.82%
TT	5.29%	4.60%	5.11%	3.41%	3.21%	3.57%	3.43%	3.47%	3.32%	3.99%
TA	4.87%	4.29%	5.00%	3.06%	2.97%	3.15%	3.08%	3.06%	2.99%	3.48%
AC	6.24%	7.32%	6.58%	7.52%	7.33%	7.49%	7.26%	7.69%	7.31%	8.11%
CA	5.25%	5.89%	5.37%	4.79%	5.30%	4.86%	5.29%	4.90%	5.36%	5.15%
TG	6.20%	5.25%	6.15%	5.54%	5.22%	5.72%	5.34%	5.52%	5.26%	5.02%
AT	3.14%	3.83%	3.22%	3.06%	3.03%	2.97%	2.98%	3.08%	3.04%	3.37%
TC	6.14%	6.83%	6.26%	6.57%	6.86%	6.44%	6.78%	6.47%	6.84%	7.10%
CT	4.21%	5.26%	4.34%	3.94%	4.43%	3.91%	4.34%	4.06%	4.48%	4.62%
GG	15.93%	10.01%	14.95%	13.14%	11.31%	13.42%	12.41%	12.94%	11.34%	10.51%
GA	5.20%	5.77%	5.12%	5.55%	5.43%	5.53%	5.44%	5.58%	5.56%	5.39%
AG	5.09%	5.62%	5.04%	5.34%	5.34%	5.42%	5.33%	5.41%	5.40%	5.23%
GT	4.00%	4.67%	3.97%	4.23%	4.31%	4.22%	4.27%	4.25%	4.36%	4.72%

**Table 4:** Downstream dinucleotide distribution

Dinucleotide	DNA NB DNA NT	DNA NB DNA TP	DNA NT DNA TP	DNA NB RNA NT	DNA NB RNA TP	DNA NT RNA NT	DNA NT RNA TP	DNA TP RNA NT	DNA TP RNA TP	RNA NT RNA TP
AA	5.15%	5.11%	5.18%	3.17%	3.23%	3.34%	3.41%	3.26%	3.33%	3.67%
TA	4.35%	4.31%	4.31%	2.45%	2.54%	2.53%	2.70%	2.51%	2.59%	2.89%
GG	10.55%	10.20%	10.50%	15.58%	14.40%	15.24%	14.17%	15.56%	14.50%	17.17%
CG	6.55%	7.40%	6.60%	13.10%	12.98%	12.62%	11.97%	12.74%	12.62%	11.28%
TT	5.25%	5.05%	5.19%	3.27%	3.24%	3.45%	3.46%	3.33%	3.29%	3.33%
TG	5.65%	5.98%	5.82%	4.37%	5.04%	4.50%	5.11%	4.49%	5.06%	4.73%
TC	5.34%	5.57%	5.61%	4.73%	5.24%	4.81%	5.27%	4.78%	5.30%	4.17%
GC	6.95%	7.68%	7.14%	8.97%	9.27%	8.79%	8.77%	8.99%	9.16%	9.22%
AT	3.47%	4.00%	3.51%	2.43%	2.51%	2.45%	2.52%	2.44%	2.50%	2.73%
CT	4.95%	5.65%	5.05%	4.20%	4.64%	4.29%	4.63%	4.24%	4.62%	3.99%
CA	5.76%	5.05%	5.59%	5.12%	4.67%	5.21%	4.86%	5.13%	4.71%	4.82%
AG	4.50%	5.29%	4.61%	4.11%	4.23%	4.05%	4.27%	4.14%	4.27%	5.04%
GT	6.71%	7.62%	7.04%	6.44%	7.00%	6.46%	6.78%	6.48%	7.10%	6.34%
CC	14.57%	9.65%	13.68%	11.98%	10.15%	12.20%	11.42%	11.84%	10.15%	9.18%
GA	6.18%	6.70%	6.33%	6.20%	6.73%	6.26%	6.63%	6.18%	6.67%	7.23%
AC	4.06%	4.73%	3.86%	3.87%	4.13%	3.82%	4.02%	3.88%	4.14%	4.22%

**Fig. 5:**
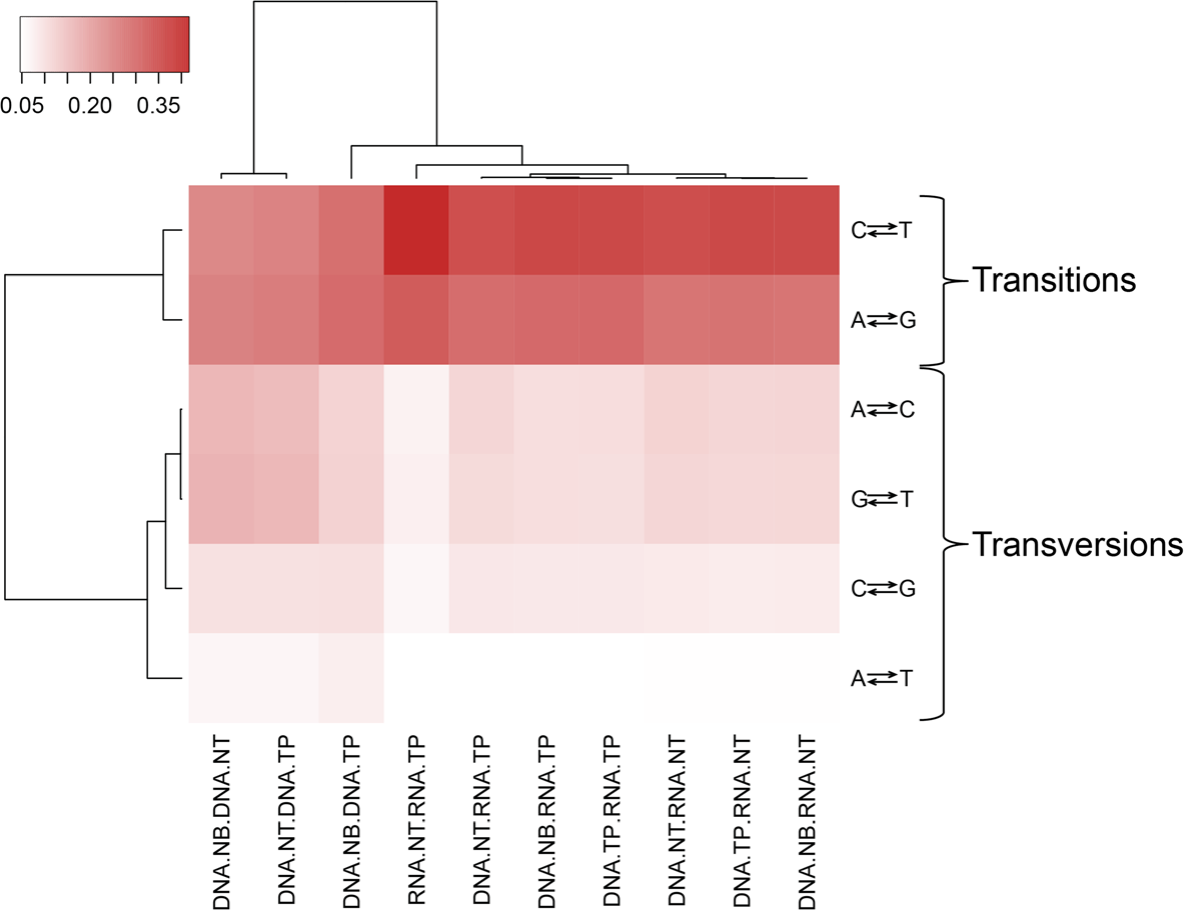
Cluster and heatmap results based on nucleotide differences between any two pairs of samples. Samples can be clearly differentiated by whether RNA is involved in the comparison. The nucleotide changes can be categorized by transversions and transitions. Transversion is heavily favored over transitions

We also performed cluster analysis using the allele change frequencies between all possible pairs of the sample-sequencing types. With the four possible nucleotides, there are six possible changes (A-C, A-T, A-G, C-T, C-G, and G-T). Similar to the cluster analysis using the frequency of two upstream and downstream nucleotides, cluster analysis showed that pairs with only DNA sequencing data form one cluster, while pairs with RNAseq data form another cluster (Fig. 5). Transition changes were clearly more preferred than transversion changes (t test *p* = 6.04E-14). The average Ti/Tv ratio of the DNA-RNA difference sites was 1.98 (range: 1.71–2.86). The Ti/Tv ratio has been shown to be strongly related to genomic region and often serves as a quality control measurement [26–28]. Our Ti/Tv ratio result suggested that the DNA-RNA differences were not random. The detailed change frequencies and analysis are presented in Table 5.

**Table 5:** Nucleotide difference between any two pairs of samples from same subject

DNA-RNA Difference	DNA NB DNA NT	DNA NB DNA TP	DNA NT DNA TP	DNA NB RNA NT	DNA NB RNA TP	DNA NT RNA NT	DNA NT RNA TP	DNA TP RNA NT	DNA TP RNA TP	RNA NT RNA TP
A-C	16.52%	11.98%	15.64%	11.70%	10.34%	12.06%	11.42%	11.52%	10.42%	7.25%
A-G	25.52%	29.60%	26.36%	27.93%	30.00%	27.87%	29.34%	28.21%	30.19%	32.46%
A-T	6.52%	7.70%	6.53%	5.21%	4.86%	5.14%	4.82%	5.25%	4.88%	4.72%
C-G	9.83%	9.87%	9.83%	8.15%	8.57%	8.39%	8.77%	8.09%	8.48%	6.33%
C-T	24.36%	28.73%	25.31%	35.70%	36.01%	35.04%	34.86%	35.81%	35.92%	41.61%
G-T	17.25%	12.11%	16.34%	11.30%	10.22%	11.51%	10.80%	11.12%	10.12%	7.63%

**Fig. 6:**
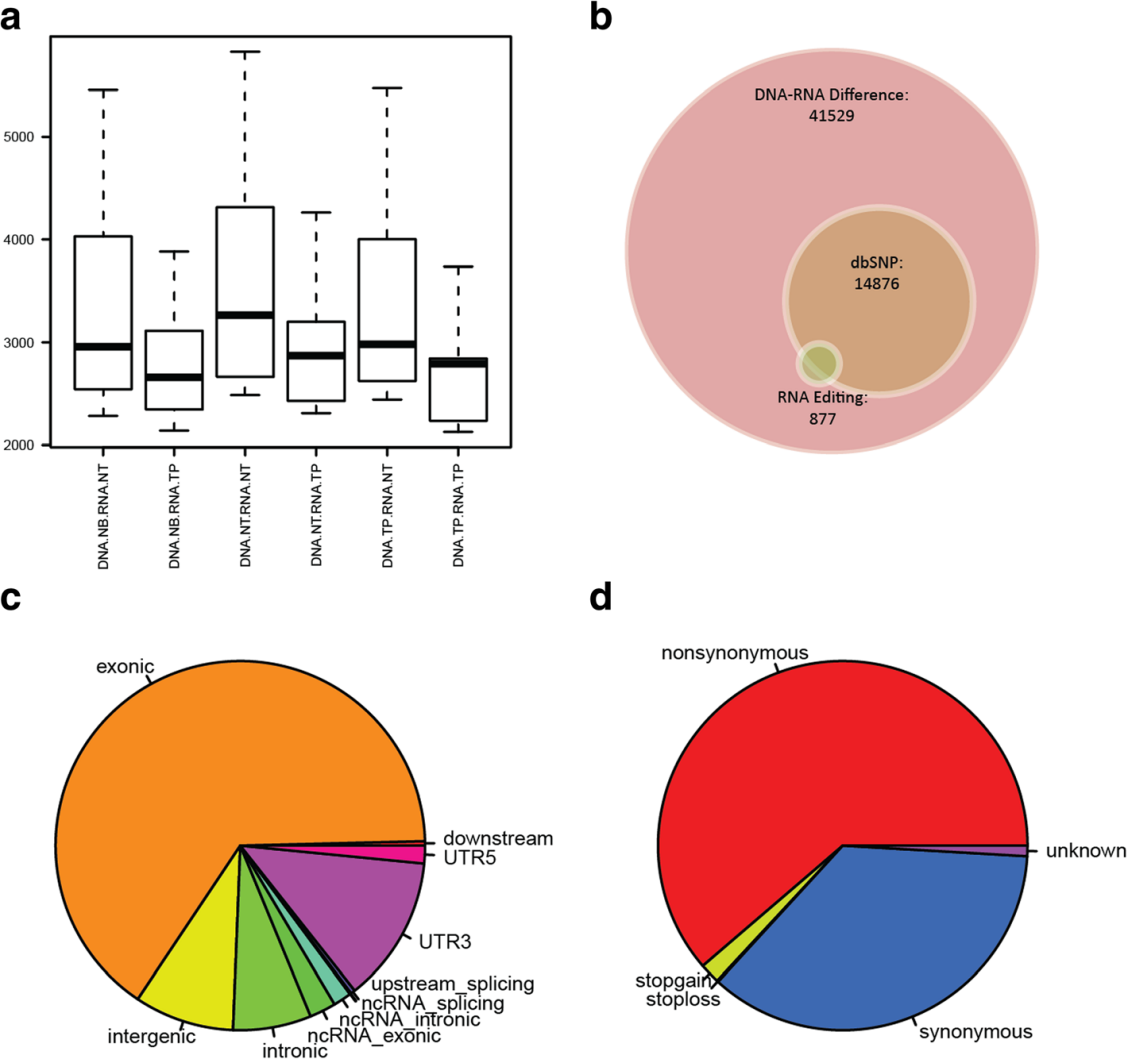
**a** Boxplots that show the number of differences between DNA and RNA. **b** There were 41,529 DNA-RNA differences, 14,876 of which were reported in dbSNP and 877 were reported in RNA editing databases. **c** Regional categorization of the DNA-RNA differences. **d** Functional categorization of the DNA-RNA differences

Lastly, we categorized the differences between DNA and RNA. The overall DNA-RNA differences per DNA-RNA pair category can be view in Fig. 6a. There are thousands of differences per category, which agrees with previous finding from Li et al. [12]. Out of all of these DNA-RNA differences, there were a total of 41,529 unique sites. Only a small portion of these sites, 877, have been documented in existing RNA editing databases; and 14,876 sites are recorded in dbSNP (Fig. 6b). Because we required both samples in the DNA-RNA comparisons to have a depth of 10 or higher, the majority of the differences are located in exonic regions (Fig. 6c, Table 6). Of these exonic differences, a majority of them (61.3%) are nonsynonymous (Fig. 6d, Table 7).

**Table 6:** Regional Categories of DNA-RNA differences

Categories	Number
downstream	160
exonic	27,073
intergenic	3623
intronic	2839
ncRNA_exonic	958
ncRNA_intronic	663
ncRNA_splicing	7
splicing	42
upstream	160
UTR3	5319
UTR5	644

**Table 7:** Functional Categories of DNA-RNA differences

Categories	Number
Nonsynonymous	16,611
Stopgain	485
Stoploss	30
Synonymous	9716
Unknown	249

## Discussion

It has been proposed that single nucleotide variants, such as SNPs and somatic mutations, can be detected using RNAseq data [8, 29]. At the same time, strong evidence demonstrates large differences of detected nucleotides between DNA and RNA sequencing data [7, 12]. To further examine the genotype differences inferred from high throughput sequencing data between DNA and RNA, and among various sample sources, we designed a study to compare the genotypes obtained from five types of high throughput sequencing data that were generated from ten individuals. With thorough analysis, we observed large differences between the genotypes inferred from DNA and RNA sequencing data, which agrees with Li et al.’s findings [12]. However, Li et al. asserts these observed differences are the true differences between DNA and RNA, not accounting for differences introduced by technical errors. The study was conducted using TCGA data. Since we do not have access to the original samples, we could not perform at qPCR validation of the DNA-RNA differences.

DNA-RNA differences can be attributed to two categorical factors: biological and technical. The biological factors can be summarized as RNA editing and polyadenylation, which are a part of the natural biological process. RNA editing is the process that results in RNA nucleotide sequences that differ from the DNA template. Polyadenylation is the addition of a Poly(A) tail to the 3′ end of the mRNA during the transcription of DNA to RNA. Technical factors include reverse transcription errors, sequencing errors, and alignment errors, which are technical difficulties that we have yet to overcome. Reverse transcription errors occur during the reverse transcription from RNA to cDNA—a mandatory step for RNAseq. Sequencing errors can result from the high throughput sequencing technology, as all types of high throughput sequencing technologies have known limits and advantages. For example, Illumina’s high throughput sequencing technology is known to be sensitive to GC content [30, 31], while 454 Life Sciences’ sequencing technology produces low quality reads with long Poly (A) and (T) tracts. Alignment errors often occur while finding the best genomic locations for a read. The current alignment algorithm is largely based on the Borrows Wheeler Transformation, an algorithm that is used in computer science to compress repeated strings that contain repeated characters. Even though alignment can happen at a global level, the human genome is too complicated and contains a vast number of homologous regions, and, alignment of RNA reads to a DNA reference sequence requires that splicing of the gene exons be taken into account. All of these factors can substantially convolute the RNA alignment process and introduce potential alignment errors. Since 2001, there have been 20 reference human genomes released. Substantial improvements have been made with each new release providing more precise descriptions of the transcriptome, which in turn increases the accuracy of alignment of RNA reads. It is possible that with further advancements to the reference human genome, we will observe fewer DNA-RNA differences.

In our analysis, we also observed differences between adjacent normal tissue and blood in DNA, which are both considered to be germline. Some of these differences can be explained by tumor contamination of the adjacent normal tissue, and/or technical errors. Our results support Gottlieb’s finding that there are potential SNP differences between normal tissue and blood [1].

## Conclusion

In conclusion, based on our analysis results, there are large differences (10%) between genotypes inferred from DNA and RNA sequencing data of the same individual. At the present time, it is difficult to assess what portion of these differences are due to biological processes and what portion of the differences are the result of technical errors. When RNAseq data is used to infer SNPs or somatic mutations, the DNA-RNA difference will result in large amounts of false positives [7], thus making RNAseq data a less than ideal source for detecting SNVs.

## Additional file

Additional file 1: Integrative Genomics Viewers screenshot of position chromosome 1:12,520,386. Alignment results for all five samples for patient A7-A0D9 are displayed. Top three are DNA samples, and bottom two are RNA samples. The reference is C. Both RNA samples detected alternative allele G, two DNA samples did not detect alternative allele G. (PNG 211 kb)
