# Supplementary figures and images for: The discrepancy among single nucleotide variants detected by DNA and RNA high throughput sequencing data
Source: BMC Genomics. 2017 Oct 3;18(Suppl 6):690. doi: 10.1186/s12864-017-4022-x (PMC5629567; doi:10.1186/s12864-017-4022-x)

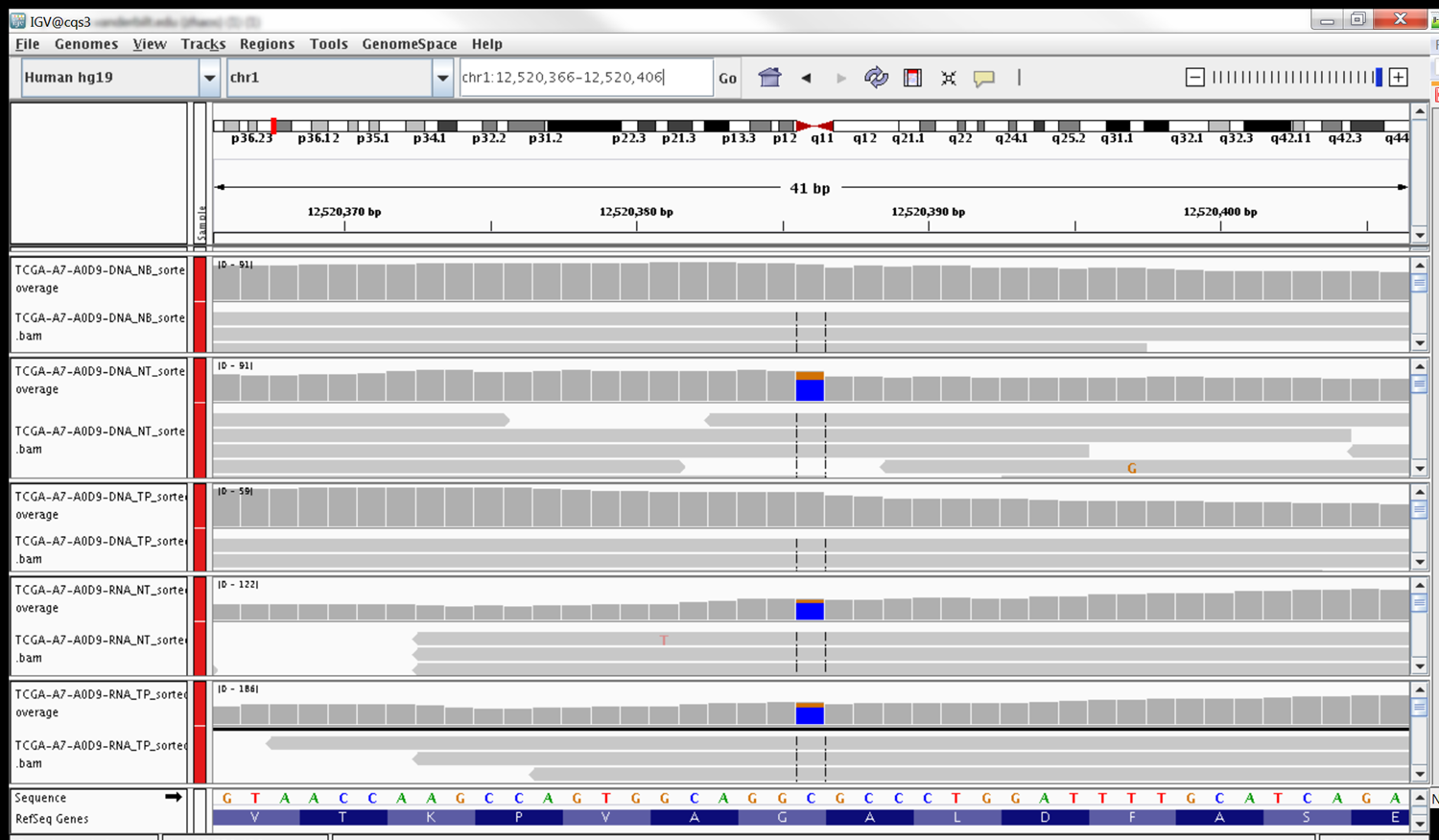

Supplement: Additional file 1: — Integrative Genomics Viewers screenshot of position chromosome 1:12,520,386. Alignment results for all five samples for patient A7-A0D9 are displayed. Top three are DNA samples, and bottom two are RNA samples. The reference is C. Both RNA samples detected alternative allele G, two DNA samples did not detect alternative allele G. (PNG 211 kb) [file 12864_2017_4022_MOESM1_ESM.png]
